# *Phestilla subodiosus* sp. nov. (Nudibranchia, Trinchesiidae), a corallivorous pest species in the aquarium trade

**DOI:** 10.3897/zookeys.909.35278

**Published:** 2020-02-05

**Authors:** Adam Wang, Inga Elizabeth Conti-Jerpe, John Lawrence Richards, David Michael Baker

**Affiliations:** 1 Chinese International School, Hau Yuen Path, Braemar Hill, Hong Kong SAR; 2 Swire Institute of Marine Science, School of Biological Sciences, The University of Hong Kong, Pok Fu Lam Road, Hong Kong SAR; 3 School of Biological Sciences, University of Hong Kong, Pok Fu Lam Road, Hong Kong SAR

**Keywords:** Nudibranchs, aquaculture, corallivore

## Abstract

*Phestilla subodiosus***sp. nov.** (Nudibranchia: Trinchesiidae) is a novel species that feeds on corals in the genus *Montipora* (Scleractinia: Acroporidae) which are economically important in the aquarium industry. Nuclear-encoded H3, 28SC1-C2, and mitochondrial-encoded COI and 16S markers were sequenced. Phylogenetic analysis, Automatic Barcode Gap Discovery (ABGD), morphological data, and feeding specialization all support the designation of *Phestilla subodiosus***sp. nov.** as a distinct species. Although new to science, *Phestilla subodiosus***sp. nov.** had been extensively reported by aquarium hobbyists as a prolific pest over the past two decades. The species fell into a well-studied genus, which could facilitate research into its control in reef aquaria. Our phylogenetic analysis also revealed *Tenellia chaetopterana* formed a well-supported clade with *Phestilla*. Based upon a literature review, its original morphological description, and our phylogenetic hypothesis, we reclassified this species as *Phestilla chaetopterana***comb. nov.**

## Introduction

While many Nudibranchia species and genera have yet to be described (Gosliner et al. 2015), the deeper relationships in the systematics of several superfamilies and families within this group have been repeatedly investigated and revised in taxonomic and systematic studies employing both morphological and molecular techniques (Wägele and Willan 2000; Carmona et al. 2013; Cella et al. 2016; Korshunova et al. 2017a, b, c, 2018a, b, 2019a, b; Martynov et al. 2019). The superfamily Fionoidea is one of these groups that was recently investigated phylogenetically with genetic markers (Wägele and Willan 2000; Cella et al. 2016). Based on a phylogenetic hypothesis and morphological reasoning, Cella et al. (2016) combined several families (Calmidae, Tergipedidae, Eubranchidae, Cuthonidae, and Trinchesiidae) into the family Fionidae, and several genera (*Catriona*, *Phestilla*, and *Trinchesia*) along with several species from *Cuthona* into the genus *Tenellia*. However, strong defining morphological characteristics were not suggested and “beyond the scope” of the study. Furthermore, one of the phylogenetic arguments put forward by Cella et al. (2016) was that because several genera formed a strongly supported clade, they should be grouped as a single genus; despite this, there were three other strongly supported early diverging subclades within this clade that were not discussed. Korshunova et al. (2017c) studied the synapomorphies of the group and determined the changes proposed by Cella et al. (2016) were under-representing ontogenetic, morphological, and ecological diversity. They resurrected several families under Fionoidea (Calmidae, Cuthonellidae, Cuthonidae, Eubranchidae, Tergipedidae, and Trinchesiidae) and several genera (*Catriona*, *Diaphoreolis*, *Phestilla*, and *Trinchesia*) under the family Trinchesiidae, which matched the subclades within the phylogeny published by Cella et al. (2016). However, even with the thorough taxonomic work being conducted, the globally distributed superfamily (Debelius and Kuiter 2007) still contains dozens of undescribed species (Gosliner et al. 2015) and at least one species, *Tenellia chaetopterana* Ekimova, Deart and Schepetov 2017, that was not incorporated in the systematic study by Korshunova et al. (2017c).

*Phestilla* (Fionidae: Trinchesiidae) was one of the genera reinstated by Korshunova et al. (2017c). The group is characterized by corallivory (Rudman 1979, 1981; Ritson-Williams et al. 2003; Faucci et al. 2007) and “the modified cerata, lacking cnidosacs but with large glandular ceratal tips” (Rudman 1981: 387). *Phestilla* represents the largest group of Nudibranchia that feed only on scleractinian corals (Ritson-Williams et al. 2003; Goodheart et al. 2017). Studies that combined morphological and molecular approaches have examined the phylogenetic relationships within *Phestilla* (Faucci et al. 2007; Cella et al. 2016; Korshunova et al. 2017c) and several *Phestilla* species have been used as model organisms for studying pharmaceutical drug targets (Kimberly 2003), larval development (Harris 1975; Haramaty 1991; Pasquinelli et al. 2000), invertebrate metamorphosis (Hadfield and Pennington 1990; Hadfield et al. 2001; Hadfield et al. 2006; Ritson-Williams et al. 2009), and predatory control of corallivores *in situ* (Gochfeld and Aeby 1997). Due to their diet, *Phestilla* nudibranchs present a challenge to coral aquaculture (D Hui, J McNelley pers. comm. 2018; Borneman 2007; Riddle 2012; Henschen 2018), often evading detection and eradication due to their small size and effective camouflage (Rudman 1979, 1982; Gochfeld and Aeby 1997).

From 2017 to 2018 we observed nudibranchs feeding on *Montipora* spp. fragments obtained from the aquarium trade in several closed system aquaria in Hong Kong. Morphological, behavioral, and genomic analysis determined that the species was previously undescribed. Later, a single specimen was obtained from the wild in Koh Tao, Thailand that was used for morphological analysis. Here, we describe this novel species of nudibranch as *Phestilla subodiosus* sp. nov. and resolve inconsistencies in the systematics of its family Trinchesiidae. *Phestilla subodiosus* sp. nov. is a corallivorous nudibranch commonly found preying on cultured corals in the genus *Montipora* (Scleractinia: Acroporidae). Aquarists report that damages caused by this species can cost hundreds of dollars (USD) per outbreak (D Hui, J McNelley pers. comm. 2018). Despite the economic and environmental importance of coral aquaculture, little information is available on the eradication and control of pest species (Borneman 2007; Riddle 2012). In the case of *Phestilla subodiosus* sp. nov. the species has not even been described despite online reports of it from as early as 2001 (Gray 2001).

## Materials and methods

### Collection and preservation

**Figure 1.**
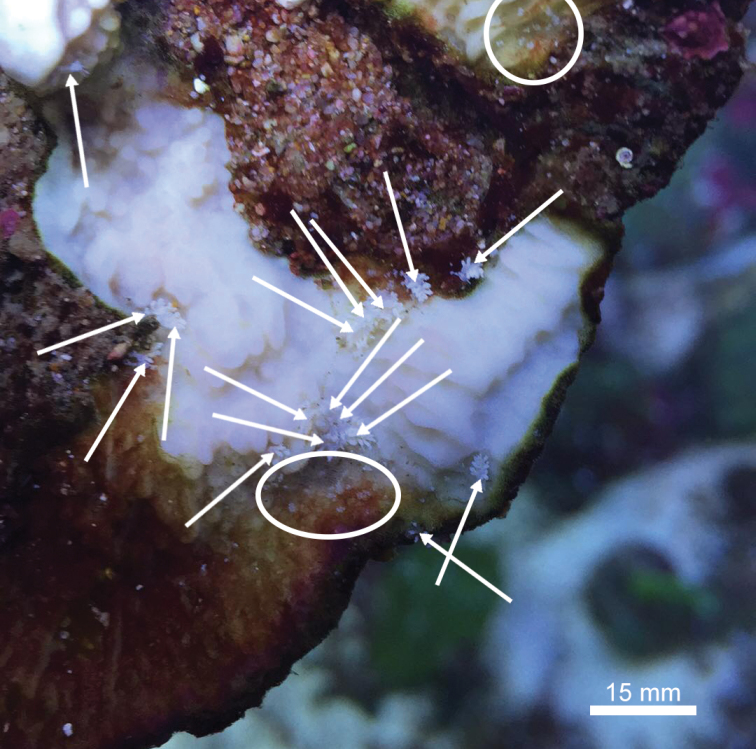
An aggregation of living individuals of *Phestilla subodiosus* sp. nov. on *Montipora* sp. White arrows indicate metamorphosed individuals; white circles indicate clusters of egg masses.

**Figure 2.**
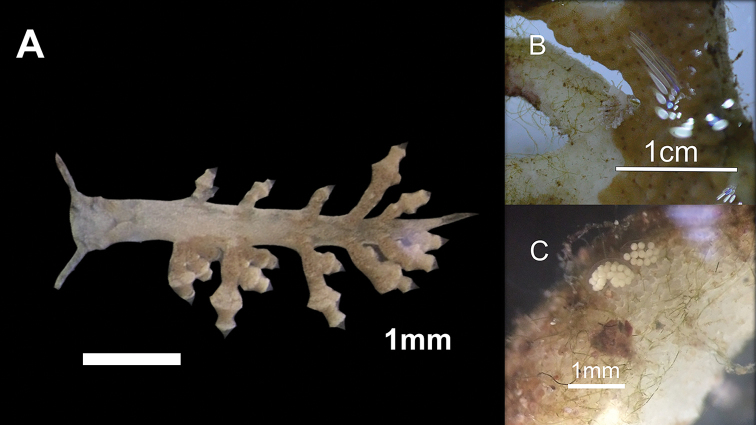
Specimens of *Phestilla subodiosus* sp. nov.: **A** adult (4 mm paratype) **B** adult feeding on *Montipora* sp. **C** paratype egg mass on *Montipora* sp. fragment.

Sexually mature nudibranchs and their egg masses were collected from *Montipora* spp. fragments (*N* > 10) between November 2017 and March 2018 (Figs 1, 2). The *Montipora* spp. fragments were either purchased from aquarium stores or obtained from other hobbyists between 2015 and 2018. A single 3 mm specimen *Phestilla subodiosus* sp. nov. was obtained from a wild locality in Koh Tao, Thailand on 22 April 2019. Adults and juveniles were relaxed for morphological analysis by the dropwise addition of 10 % magnesium chloride and fixed in formalin for 24 hours before being preserved in 95 % ethanol. Egg masses and specimens for DNA extraction were fixed in 95% ethanol directly after collection.

### DNA extraction and amplification

Total genomic DNA was extracted from six specimens using the DNeasy blood and tissue extraction kit (Qiagen, Germany), following the manufacturer’s protocol. Four loci were amplified with Polymerase Chain Reaction (PCR): mitochondrial Cytochrome *c* oxidase subunit I (COI), mitochondrial 16S structural rRNA subunit (16S), nuclear Histone H3 (H3), and nuclear 28S structural rRNA subunit (28S). Primers used are listed in Table 1. PCR reactions were conducted in 20 µl volume reactions, containing 2 µl of the forward and reverse primers (10 µM concentration) and extracted DNA, 6 µl of nuclease-free water, and 8 µl of PCR MasterMix (Sigma-Aldrich) or Hot Start Taq DNA Polymerase (BiotechRabbit). Amplification of the COI and 28S markers was performed with an initial denaturation of 5 minutes at 94 °C, followed by 35 cycles of denaturing for 1 minute at 94 °C, annealing for 30 seconds at 42.5 °C for COI and 45 °C for 28S, and elongation for 1 minute at 72 °C, with the final elongation for 7 minutes at 72 °C. Amplification for H3 was performed with an initial denaturation for 3 minutes at 94 °C, followed by 35 cycles of denaturation for 35 seconds at 94 °C, annealing for 1 minute at 50 °C, and elongation for 1 minute at 72 °C, with the final elongation for 7 minutes at 72 °C. Amplification for 16S was performed with an initial denaturation of 3 minutes for 94 °C, 39 cycles of denaturation for 30 seconds at 94 °C, annealing for 30 seconds at 52.5 °C, and elongation for 1 minute at 72 °C, with the final elongation for 5 minutes at 72 °C. All reactions were performed on a Veriti Thermal Cycler (Applied Biosystems, USA). Amplified products were visualized on a 2% agarose gel prior to sequencing.

**Table 1.** Primers used for PCR and sequencing of *Phestilla subodiosus* sp. nov.

LCO 1490	5’-GGTCAACAAATCATAAAGATATTGG-3’	(Folmer et al. 1994)	5 min at 94 °C, 35× [1min at 94 °C, 30s at 42.5 °C, 1min at 72 °C], 7 min at 72 °C
COIH-2	5’-TAYACYTCRGGATGMCCAAAAATCA-3’	(Cella et al. 2016)	
H3AF	5’-ATGGCTCGTACCAAGCAGACVGC-3’	(Colgan et al. 1998)	3min at 94 °C, 35× [35s at 94 °C, 1min at 50 °C, 1min at 72 °C], 7min at 72 °C
H3AR	5’-ATATCCTTRGGCATRATRGTGAC-3’	(Colgan et al. 1998)	
16S arL	5’-CGCCTGTTTAACAAAAACAT-3’	(Palumbi et al. 2002)	3min at 94 °C, 39× [30s at 94 °C, 30s at 50–55 °C, 1min at 72 °C], 5min at 72 °C
16S R	5’-CCGRTYTGAACTCAGCTCACG-3’	(Puslednik and Serb 2008)	
28SC1	5’-ACCCGCTGAATTTAAGCAT-3’	(Dayrat et al. 2001)	5min at 94 °C, 35× [1min at 94 °C, 30s at 45 °C, 1min at 72 °C], 7min at 72 °C
28SC2	5’-TGAACTCTCTCTTCAAAGTTCTTTTC-3’	(Le et al. 1993)	

PCR products for COI, 28S, and H3 were purified with ExoSAP-IT™ PCR Product Cleanup Reagent (ThermoFisher, USA) and cycle sequenced using the BigDye Terminator v3.1 Cycle Sequencing Kit (ThermoFisher, USA), both in accordance with the manufacturer’s instructions. Sequencing was performed on an ABI 3130xl Genetic Analyzer (ThermoFisher, USA). 16SPCR products were sequenced externally by the Beijing Genomics Institute (Shenzhen, China).

### Phylogeny

Raw reads obtained from *Phestilla subodiosus* sp. nov. were assembled and edited visually with Geneious 11.1.4 (Kearse et al. 2012). nBLAST (Altschul et al. 1990) searches revealed that significantly similar H3, 16S, and COI sequences were available, while few were available for 28S. Due to the lack of similar 28S sequences, this locus was ultimately not used in the phylogenetic analysis. COI, 16S, and H3 sequences (*N* = 141) of 47 species, including 9 undescribed species, from eight Fionoidea families (Suppl. material 1: Table S1), were downloaded from NCBI’s GenBank (Clark et al. 2016). COI, 16S, and H3 sequences were aligned using MUSCLE (Edgar 2004) and trimmed to 658 bp, 492 bp, and 328 bp, respectively, using MEGA X (Kumar et al. 2018). GUIDANCE-2 (Sela et al. 2015) was employed to identify offending sequences in alignments. Hypervariable indel-rich regions in the 16S gene were not removed from the analysis (Cella et al. 2016). Sequences were concatenated manually using MEGA X (Kumar et al. 2018).

IQ-Tree (Nguyen et al. 2015) was used to infer evolutionary histories using the Maximum Likelihood (ML) method with a partitioned analysis (Chernomor et al. 2016) and 1500 pseudoreplicates using the bootstrap method to estimate the ML support values (BS). IQ-Tree’s ModelFinder tool (Kalyaanamoorthy et al. 2017) invoked a full tree search of every model for each partition to calculate the Bayesian Information Criterion (BIC), Akaike Information Criteria (AIC), and Corrected Akaike Information Criteria (CAIC) of each substitution model. Based upon BIC, TVM+F+I+G4 for COI and 16S, and TIM2+F+I+G4 for H3 were used for the phylogenetic analysis. MrBayes (Ronquist et al. 2012) was used to infer another evolutionary history using Bayesian Inference (BI) with the GTR+I+G model. Two simultaneous Metropolis-Coupled Monte Carlo Markov Chains (MCMCMC) were run with four chains – one cold and three hot (temp = 0.1) – for 6,000,000 generations. The prior was flat Dirichlet. Diagnostics were calculated every 5000 generations with a 25% burn-in to calculate Posterior Probability (PP). Cold chains were sampled every 1000 generations. Raw newick files were reformatted using MEGA X (Kumar et al. 2018). Final trees were edited and annotated using Photoshop CC 2017 (Adobe, USA).

Trees for each individual gene were computed to gain a better understanding of the systematics of the group. ML trees were estimated using IQ-Tree (Nguyen et al. 2015) with 10,000 bootstrap pseudoreplicates using the UFBoot2 Method (Hoang and Chernomor 2017) and models were automatically found using IQ-Tree’s ModelFinder tool (Kalyaanamoorthy et al. 2017). The models utilized were TVM+F+I+G4 for COI and 16S, and TIM2+F+I+G4 for H3. BI trees were estimated using MrBayes (Ronquist et al. 2012) with the GTR+I+G model. Two simultaneous MCMCMC with a flat Dirichlet prior were run for 3,000,000 generations using three hot (temp = 0.1) and one cold chain, with diagnostics being calculated every 1000 generations, a 25% burn-in and cold chain sampling every 500 generations.

### Species delineation

An online version of the Automatic Barcode Gap Discovery (ABGD) program (Puillandre et al. 2012) was employed to delineate species using a dataset of 15 *Phestilla*COI sequences from eleven species (Suppl. material 1: Table S1). The ABGD settings were: P_min_ = 0.001, P_max_ = 0.1, Steps = 10, X = 1.5, Nb bins = 20. Three different distance models, Jukes-Cantor (JC69), Kimura (K80) TS/TV 2.0, and Simple Distance, were run (Puillandre et al. 2012). Uncorrected pairwise distances (*p*-distance) for COI were calculated in MEGA X (Kumar et al. 2018) with the nucleotide substitution type using the same *Phestilla*COI dataset. The rate variation among sites was modelled with a gamma distribution (shape parameter = 4) with invariant sites (G+I). All ambiguous positions were removed for each sequence pair. The bootstrap method with 10,000 pseudoreplicates was used to estimate variance.

### Morphological analysis

Live adult (4 mm paratype) and juvenile individuals (1–3 mm paratypes) were photographed using a Nikon D5100 camera (Nikon, Japan) with AF-S Nikkor 18–55 mm 1:3.5–5.6G lens (Nikon, Japan). The holotype (2 mm) and a paratype (egg mass) were imaged using a Leica DFC295 microscope camera (Germany) with a 0.63X Stereo Microscope C-Mount (Leica, Germany) to examine external structures. The holotype obtained from captive *Montipora* spp. (2 mm) and the paratype collected from Thailand (3 mm) were dissected to isolate the buccal mass and reproductive system. Buccal mass was dissolved in dilute bleach (~ 1:30) to review radula and jaw plates. Radula, jaw plates, and reproductive system were imaged and examined under a Meiji Techno M1510 Trinocular Compound Microscope (Meiji Techno Co., Japan). Images were edited and annotated using Photoshop CC 2017 (Adobe, US). All type material was deposited at the Museum of The Swire Institute of Marine Science at The University of Hong Kong.

### Observed host species

To elucidate the possible coral hosts of *Phestilla subodiosus* sp. nov., preliminary data of observed hosts were recorded. Individuals of *Phestilla subodiosus* sp. nov. (5 ≥ *N* ≥ 10) and a single fragment of one of eight species of coral (Table 2) were isolated in a glass beaker (50 ml) for a week. Coral species were identified according to Veron (2000), Chan et al. (2005), and Wallace et al. (2012), and several species representing a diverse selection of colony morphologies and coenosteum phenotypes were chosen (Table 2). Temperature was maintained constant by partially submerging the jars into a water bath 24–27 °C, and approximately 75% of the water was changed daily. A coral species was counted as a host species if they fulfilled two criteria: firstly, *Phestilla subodiosus* sp. nov. had to form an aggregation (see Fig. 1) within 3–4 cm of the coral (Morton et al. 2002); and secondly, the coral had to show evidence of tissue loss from predation surrounding the aggregations (Figs 1, 2B; Ritson-Williams et al. 2003, Dalton and Godwin 2006).

**Table 2.** Observational data of the feeding preference of *Phestilla subodiosus* sp. nov. Abbreviations: N indicates that this species of coral did not satisfy the two conditions needed to be counted as a host coral; Y indicates that the species did satisfy both conditions needed to be counted as a host coral.

Family	Genus	Species	Growth form	Host species
Acroporidae	* Acropora *	* samoensis *	Digitate corymbose. Thick branches.	N
		* pruinosa *	Digitate arborescent. Thin branches.	N
	* Montipora *	sp. 1	Encrusting.	Y
		sp. 2	Digitate arborescent. Thin branches.	Y
		sp. 3	Laminar scrolling.	Y
		sp. 4	Laminar scrolling or encrusting.	Y
Lobophylliidae	* Echinophyllia *	* aspera *	Laminar scrolling or encrusting.	N
Poritidae	* Porites *	sp. 1	Encrusting.	N

## Results

### Sequence analysis

In total, 17 of 24 sequences obtained from six sexually mature individuals were used for the final analysis: five from COI, two from 16S, four from 28S, and six from H3. GUIDANCE-2 revealed that the 16S sequence of *Eubranchus rustyus* was low quality and thus it was removed from the alignment. The concatenated dataset used in the phylogenetic analysis was 1255 bp (549 bp for COI, 379 bp for 16S, 327 bp for H3) long, including indels. Trees generated for each individual gene dataset (Suppl. material 2: S2) support the resolution hierarchy proposed by Cella et al. (2016).

**Figure 3.**
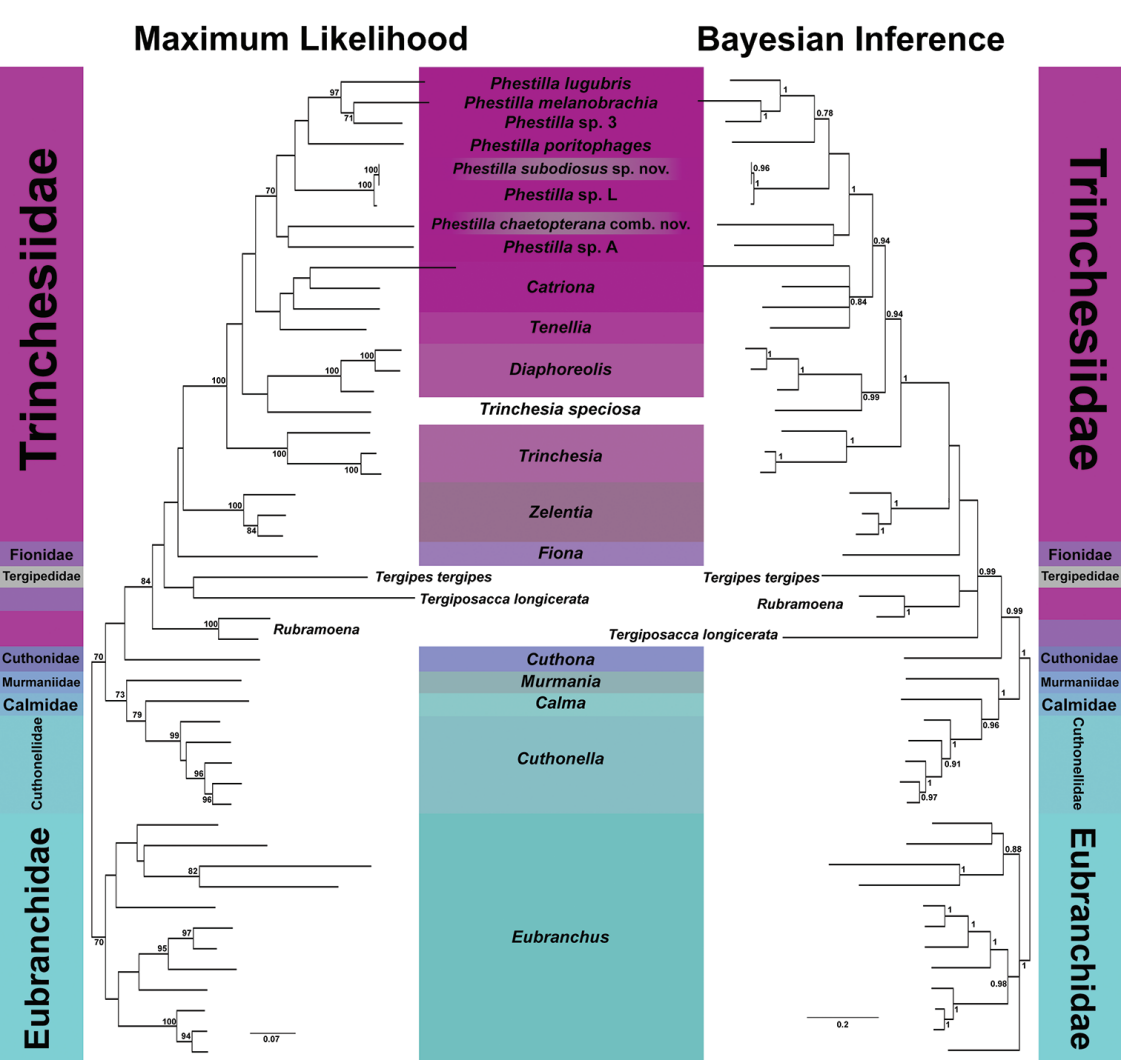
Combined COI-16S-H3 Maximum Likelihood and Bayesian Inference phylogenetic hypotheses. Support values indicate Bootstrap (BS) and Posterior Probability (PP) rounded to two significant digits on the ML and BI trees. *Phestilla subodiosus* sp. nov. and *P. chaetopterana* comb. nov. are highlighted. Trees rooted on *Eubranchus*.

The ML and BI phylogenic hypotheses (Fig. 3) and the tree published in Cella et al. (2016) resolved with similar topologies; however, none of the trees were congruent on the relationship between *Rubroamoena*, *Tergipes*, and *Tergiposacca*. These differences could be attributed to the fact that the ML and BI analyses used different models. While in theory the general topology of the trees produced should be the same since the search space of GTR encompasses the spaces of TVM and TIM2, algorithms that maximize likelihoods are prone to getting stuck on a local optimum, especially with constrained parameters or small sample sizes (Hillis et al. 1996). Further research is required for the field of nudibranch systematics to decide which model to trust. However, this does not explain the recovery of *Trinchesia* as polyphyletic in both trees, with *Tr. speciosa* forming a clade with *Diaphoreolis* (BS = 54%, PP = 0.99). The families Trinchesiidae, Fionidae and Tergipedidae were also recovered as paraphyletic and polyphyletic in both trees. Further research is required to identify whether these were artefacts of unbalanced taxon sampling or indicative of flawed taxonomic grouping. However, both trees did recover described *Phestilla* species and *P.* sp. 3 as monophyletic (BS = 54%, PP = 0.78), forming clades with *Phestilla subodiosus* sp. nov. and *P.* sp. L (BS = 64%, PP = 0.65), and with *Te. chaetopterana* and *P.* sp. A (BS = 70%, PP = 1). The clade containing *Phestilla subodiosus* sp. nov. and *P.* sp. L had very short branch lengths and had high support values (BS = 100%, PP = 1), suggesting that *Phestilla subodiosus* sp. nov. and *P.* sp. L are the same species. Both trees also recovered *Tenellia* as polyphyletic, with *Te. chaetopterana* in the same clade as *Phestilla*. To solve this issue, *Te. chaetopterana* should be transferred to *Phestilla*, or to a new genus with *Phestilla* sp. A.

Pairwise distances (Table 3) based on the COI dataset revealed that all *Phestilla subodiosus* sp. nov. samples had virtually identical COI sequences (*p* = 0.0% ± 0.0 %). *Phestilla subodiosus* sp. nov. was most closely related to *P.* sp. L (*p* = 1.0% ± 1.4%) and *P.* sp. 3 (*p* = 1.2% ± 1.4%). All other species had *p* > 11.0%, providing more evidence that *P.* sp. L is the same species as *Phestilla subodiosus* sp. nov. The next closest species to *Phestilla subodiosus* sp. nov. were *P.* sp. 1 (*p* = 11.8% ± 1.3%), *P. poritophages* (*p* = 14.2% ± 1.4%), and *P. minor* (*p* = 14.6% ± 0.4%). The analysis revealed that *P. lugubris* and *P. sibogae* had very similar COI sequences (*p* = 2.2% ± 1.6%), providing evidence for their synonymy.

**Table 3.** Uncorrected COI*p*-distances (%) among all species of described *Phestilla* with available sequences. Percentages all rounded to one decimal place. Standard error (%) estimates rounded to one decimal place generated from bootstrapping (*N* = 10,000) are shown above the diagonal.

		**1**	**2**	**3**	**4**	**5**	**6**	**7**	**8**	**9**	**10**	**11**	**12**	**13**	**14**	**15**
**1**	*Phestilla minor*		1.6	1.4	1.4	1.4	1.4	1.4	1.4	1.6	1.6	1.4	1.4	1.4	1.3	1.4
**2**	*P. sibogae*	20		1.5	1.5	1.5	1.5	1.5	1.5	1.7	0.6	1.6	1.6	1.5	1.5	1.5
**3**	*P.* sp. 2	15.2	18.9		1.4	1.4	1.4	1.4	1.4	1.6	1.5	1.3	1.5	1.4	1.4	1.3
**4**	*P. subodiosus* sp. nov. PS1	14.6	17.5	13.7		0.0	0.0	0.0	0.0	1.5	1.4	1.4	1.4	0.4	1.3	0.4
**5**	*P. subodiosus* sp. nov. PS3	14.6	17.5	13.7	0.0		0.0	0.0	0.0	1.5	1.4	1.4	1.4	0.4	1.3	0.4
**6**	*P. subodiosus* sp. nov. PS4	14.6	17.5	13.7	0.0	0.0		0.0	0.0	1.5	1.4	1.4	1.4	0.4	1.3	0.4
**7**	*P. subodiosus* sp. nov. PS5	14.6	17.5	13.7	0.0	0.0	0.0		0.0	1.5	1.4	1.4	1.4	0.4	1.3	0.4
**8**	*P. subodiosus* sp. nov. PS6	14.6	17.5	13.7	0.0	0.0	0.0	0.0		1.5	1.4	1.4	1.4	0.4	1.3	0.4
**9**	*P. chaetopterana* comb. nov.	17.1	18.4	17.1	14.9	14.9	14.9	14.9	14.9		1.6	1.6	1.5	1.5	1.5	1.5
**10**	*P. lugubris*	19.9	2.2	18.7	16.7	16.7	16.7	16.7	16.7	17.8		1.6	1.5	1.4	1.5	1.4
**11**	*P. melanobrachia*	14.6	19.7	12.6	15.9	15.9	15.9	15.9	15.9	19.3	19.6		1.5	1.4	1.4	1.4
**12**	*P. poritophages*	14.2	18.5	16.7	14.2	14.2	14.2	14.2	14.2	15.8	17.7	17.6		1.4	1.4	1.4
**13**	*Phestilla* sp. L	14.1	16.9	13.6	1.0	1.0	1.0	1.0	1.0	14.7	16.1	15.9	14.1		1.3	0.5
**14**	*Phestilla* sp. 1	12.9	18	14.6	11.8	11.8	11.8	11.8	11.8	15.5	17.7	14.9	15.1	11.8		1.3
**15**	*Phestilla* sp. 3	13.7	17.4	13.2	1.2	1.2	1.2	1.2	1.2	14.5	16.9	15.8	14.1	1.5	11.6	

All three ABGD models elucidated ten partitions: simple distance found ten partitions with eight groups; while JC69 and K80 found five partitions with eight groups and five partitions with ten groups. In the partitions with eight groups, *Phestilla subodiosus* sp. nov., *P.* sp. L, and *P.* sp. 3 as well as *P. lugubris* and *P. sibogae* were grouped together. This provides additional evidence that *Phestilla subodiosus* sp. nov., *P.* sp. L, and *P.* sp. 3 are the same species and that *P. lugubris* and *P. sibogae* are synonymous. However, in the partitions with ten groups, while *P. lugubris* and *P. sibogae* were grouped together, *Phestilla subodiosus* sp. nov. was distinct to *P.* sp. L and *P.* sp. 3. These partitions are likely statistical anomalies due to the oversampling of virtually identical *Phestilla subodiosus* sp. nov. sequences.

### Observed host species

Of all the coral species examined (Table 2), only *Montipora* species qualified as a suitable host. In all the other trials, *Phestilla subodiosus* sp. nov. wandered across the containment capsules and neither host criteria were met. These results indicate that prey choice is independent to host coral colony morphology. However, it is worthwhile to note that the *Acropora samoensis* specimen did suffer tissue loss towards the base and began re-encrusting within a week after the experiment ended, indicating that the specimen was in fact healthy. It is unclear if the tissue loss was due to predation from *Phestilla subodiosus* sp. nov, or an adverse reaction to another factor.

## Taxonomic account

### Order Nudibranchia

#### Superfamily Fionoidea Gray, 1857

**Family Trinchesiidae Nordsieck, 1972**

##### Genus Phestilla

Taxon classificationAnimaliaNudibranchiaTrinchesiidae

Bergh, 1874

CFC50CE8-D2A0-5A99-972C-CF179D500F14

###### Diagnosis.

“Physical form quite depressed. An edge anterior to the head, wing-like, attached to [...]; oral tentacles short, rhinophores simple. Cerata arranged on singular slanting rows, lacking cnidosacs. [...] Masticatory edge contains mandibles behind teeth (round, with irregular serration). Radula uniserial.” – Bergh, 1874: 1, partially translated.

###### Included species.

*Phestilla chaetopterana* (Ekimova, Deart & Schepetov, 2017), comb. nov., *Phestilla lugubris* (Bergh, 1870), *Phestilla melanobrachia* (Bergh, 1874), *Phestilla minor* (Rudman, 1981), *Phestilla panamica* (Rudman, 1982), *Phestilla poritophages* (Rudman, 1979), *Phestilla subodiosus* sp. nov.

###### Remarks.

Historically, *Phestilla* was placed in the family Tergipedidae. This family contained a large “unnecessary and unnatural” number of genera (Rudman 1979: 344). Phylogenetic analysis revealed that this grouping was polyphyletic and a “radical solution” (Cella et al. 2016: title) was proposed: several families were combined into the family Fionidae, and several genera, including *Phestilla*, into the genus *Tenellia* (Cella et al. 2016). However, a study into the ontogeny of these groups elucidated that Cella et al.’s (2016) taxonomic decisions were underrepresenting the molecular, ecological, morphological, and ontogenetic diversity of the clades; thus, the families and genera that were combined into Fionidae and *Tenellia* were reinstated (Korshunova et al. 2017c). While there is controversy surrounding which interpretation is the taxonomic truth, we have designated *Phestilla* as a separate genus to *Tenellia* based on the arguments presented by Korshunova et al. (2017c). However, given the results of the *p*-distance and ABGD analysis, we follow Cella et al. (2016) and Rudman’s (1981) decisions to synonymize *P. sibogae* with *P. lugubris*.

At the same time that Korshunova et al. (2017c) published their findings, Ekimova et al. (2017) published a paper describing *Tenellia chaetopterana*, a species that clusters phylogenetically and morphologically with *Phestilla*. As both papers were released on the same date (26 September 2019), Ekimova et al. (2017) were unable to incorporate the revised designations from Korshunova et al. (2017c) into their description. However, there are considerable differences between *Te. chaetopterana* and the other *Phestilla* species. Firstly, the radular cusp and lateral denticle proportions are unique in the entire family (Korshunova et al. 2017c), but the general pattern is similar. Secondly, the species lacks penile glands or bulbs. Thirdly each ceratal row only has a single cerata (Ekimova et al. 2017). Finally, *Te. chaetopterana* would represent the first *Phestilla* species that does not feed on scleractinian corals (Rudman 1979, 1981, 1982; Goodheart et al. 2017). Further research is required to determine whether *Te. chaetopterana* should represent a new genus or another species of *Phestilla*. Based on our independent phylogenetic analysis and the synapomorphies shared by *Te. chaetopterana* and *Phestilla*, we propose transferring *Te. chaetopterana* to the genus *Phestilla* as the most parsimonious solution.

##### Phestilla subodiosus sp. nov.

Taxon classificationAnimaliaNudibranchiaTrinchesiidae

6F121D2D-FAB4-58F7-B5A1-446F71F20B07

http://zoobank.org/F5F4BF04-1295-4A66-87F9-F09BC61590EB

**Figure 4.**
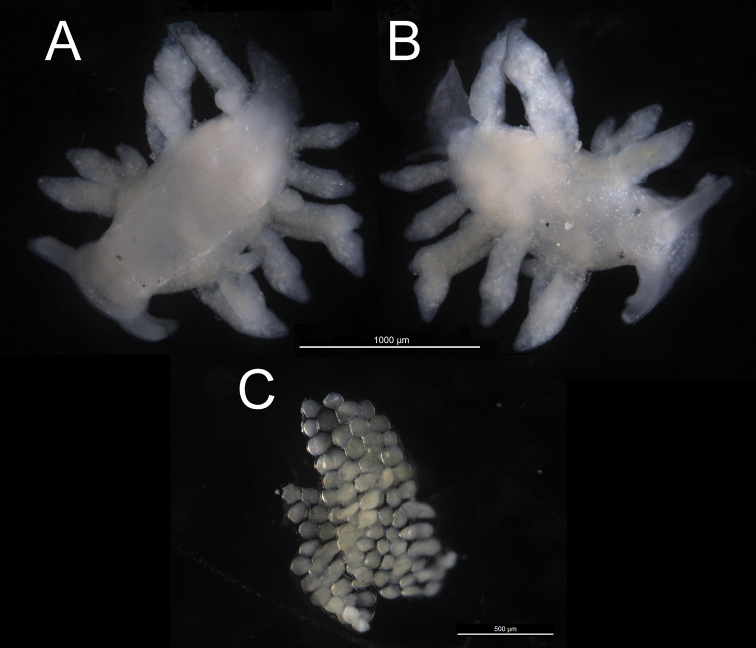
Preserved holotype 2 mm and eggs of *Phestilla subodiosus* sp. nov.: **A** ventral view of holotype **B** dorsal view of holotype **C** preserved egg cluster paratype collected from *Montipora* sp. fragment.

Figures 1 , 2 , 3 , 4

Tenellia sp. L: Cella et al. 2016: 9, 14, fig. 2, table 5 (locality unlisted).
Tenellia sp.: Cho et al. 2018: GenBank Accession number MG878397 (Jeju Island, South Korea).

###### Type Material.

**Figure 5.**
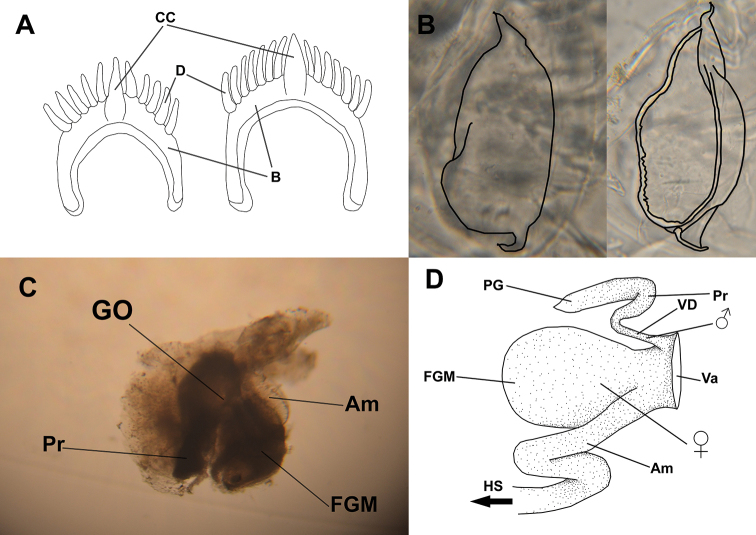
Internal morphology of *Phestilla subodiosus* sp. nov.: **A** schematic of rachidian tooth. Abbreviations: B, base; D, denticles; CC, central cusp **B** schematic of jaw plates overlaid onto microscope imagery **C** microscope imagery of reproductive system. Abbreviations: GO, genital opening; Pr, prostate; FGM, female gland mass; Am, ampulla **D** schematic of reproductive system. Abbreviations: PG, penile gland; Pr, prostate; VD, vas deferens; Va, vagina; FGM, female gland mass; Am, ampulla; HS, hermaphrodite system.

***Holotype***: [SWIMS-MOL-17-001]. 1 specimen 2 mm long in 95% ethanol, dissected, Hong Kong SAR: *Montipora* spp., cultured in aquaria, coll. A. Wang, 19 Nov. 2017 (Figs 4a, b, 5c).

***Paratypes***: [SWIMS-MOL-17-002]. 1 egg case 1 mm long in 95% ethanol. Hong Kong SAR: *Montipora* spp., cultured in aquaria, coll. A. Wang, 25 Nov. 2017 (Figs 2c, 4c).[SWIMS-MOL-18-001]. 1 specimen 1.2 mm long in 95% ethanol. Hong Kong SAR: *Montipora* spp., cultured in aquaria, coll. A. Wang, 8 Mar. 2018 (live specimen in Fig. 1). [SWIMS-MOL-19-008]. 1 specimen 3.0 mm long in 95% ethanol, dissected, Thailand: Koh Tao, Taa Chaa, depth 5 m, coll. R. Mehrotra, 22 Apr. 2019 (Fig. 5b) [SWIMS-MOL-17-003]. DNA extract from whole specimen 4mm long in 100% ethanol. Hong Kong SAR: *Montipora* spp., cultured in aquaria, coll. A. Wang, 19 Nov. 2017 (Fig. 2a) [SWIMS-MOL-18-002], [SWIMS-MOL-18-003], [SWIMS-MOL-18-004], [SWIMS-MOL-18-005], [SWIMS-MOL-18-006], [SWIMS-MOL-18-007]. DNA extracts from whole specimens 1–3.5 mm long in 100% ethanol. GenBank, respectively, Hong Kong SAR: *Montipora* spp., cultured in aquaria, coll. A. Wang, 8 Mar. 2018 (live specimens in Fig. 1).

###### Etymology.

The specific epithet, *subodiosus*, Latin for odious and vexatious, is symbolic of its status as a pest in the aquarium trade, and also a homage to the time and prized *Montipora* colonies the first author lost to in an outbreak of this species.

###### Distribution.

Specimen collected from Koh Tao, Thailand (this paper). Reported from Jeju Island, Korea (Cho et al. 2018 as *Tenellia* sp.) and confirmed using molecular methods. A similar species reported from Singapore according to a personal communication with Harris published by Robertson (1987: 3), unconfirmed. The type locality of the material from Cella et al. (2016) was not listed.

###### Description.

***External morphology*** (Figs 1, 2, 4). Thin elongate body. Sexually mature adults 1.5 mm to 4 mm in length, 0.5 mm to 1 mm in width. Oral tentacles connected to oral veil arising from edge approximately under rhinophores, brown band near the distal third. Rhinophores rounded distally, not distinct and lacking lamellae, with brown band near middle. Oral tentacles and rhinophores approximately same length. Eyes slightly posterior to each rhinophore. Body lacking obvious rhinophoral sheaths. First ceratal row slightly posterior to rhinophores. Fully developed rows contain three cerata. Cerata unbranching and arranged regularly in sloping transverse rows with two to three rows adjoining pericardium. One to three rows of cerata anterior to pericardium with no precardiac rows. Cerata lacking cnidosacs and always swollen terminally. Two to three additional swollen bulbs on fully developed cerata (Fig. 2). Pericardium hump thick in relation to rest of body, nearly 1 mm thick, beginning at first cerata row and ending between second and third row (Fig. 2). Body tapers strongly in thickness (<< 1 mm) after pericardium hump. Gonopore below and anterior to first cerata row, approx. at same height as the second cerata on the first row. Mouth large, diameter nearly equal to width of body, and clearly separated from foot.

***Internal morphology*** (Fig. 5). Jaws translucent and thin, smaller than 0.5 mm in 3 mm individual. Radular formula 12 × 0.1.0 in 3 mm individual, uniseriate. Teeth with central cusp and five to seven denticles on each side. Denticles and cusp arranged on curved edge. All denticles approx. same length. Central cusp longer and reaching slightly further than innermost denticles. Lacking secondary denticles. Reproductive system diaulic and spread throughout body. Penile bulb curved, connected to genital opening by short prostate, and adheres to wall of nudibranch. Female gland mass diameter 1.5 times size of penile bulb. Ampulla long and winding, diameter slightly larger than that of penile bulb, connected to vagina and appressed onto female gland mass, leading to hermaphrodite system. Lacks vas deferens. Penile bulb, female gland mass, and ampulla 0.5 mm to 1 mm combined.

***Color.*** Two ontogenetic color forms. Juvenile animals with white epidermal pigment throughout entire body. Adults with white epidermal pigment and translucent ceratal epidermis. Cerata speckled with brown clots, possibly from internal fluids or dinoflagellates of Symbiodiniaceae from coral hosts. Swollen regions on cerata lack speckles. Speckle density decreases towards the posterior of the cerata.

***Defense mechanisms*.** Cerata observed to autotomize and secrete viscous adhesive mucus, usually encapsulating abscised ceras, when animal is disturbed tacitly.

***Observed prey items.*** Preys on coral species in the genus *Montipora*. Does not feed on corals of genera *Porites*, *Acropora*, and *Echinophyllia*. Reports of feeding on corals in genus *Anacropora* (Henschen 2018), a sister genus to *Montipora* (Fukami et al. 2000); however, this observation is unconfirmed by the authors.

## Taxonomic remarks

Based solely on the morphological key given in Korshunova et al. (2017c), *Phestilla subodiosus* sp. nov. does not fit in any of the genera of Trinchesiidae. They defined *Phestilla* as lacking an oral veil, while it was evident that *Phestilla subodiosus* sp. nov. had one. However, in both the original descriptions and redescriptions of various *Phestilla* species in Rudman (1979, 1981, 1982), oral veils were present. Bergh’s (1874: 1) original description of *Phestilla* also referred to an “edge anterior to the head”, which is likely an oral veil. It is therefore appropriate to place *Phestilla subodiosus* sp. nov. in this genus.

Morphologically, *Phestilla subodiosus* sp. nov. is most similar to *P. minor* and *P. poritophages* in color forms and swollen cerata, but is distinguished by several characters: firstly, *adult Phestilla subodiosis* sp. nov. we observed averaged 3.5 mm in length, approximately half of the size *P. minor* (Rudman 1981) and *Phestilla poritophages* (Rudman 1979); secondly, *Phestilla subodiosus* sp. nov. only has three cerata per row, while *P. minor* has four to five (Rudman 1981) and *Phestilla poritophages* has four (Rudman 1979); thirdly, *Phestilla subodiosus* sp. nov. has only two rows of cerata anterior to its pericardium, while both *P. minor* and *P. poritophages* have three (Rudman 1979, 1981); fourthly, *Phestilla subodiosus* sp. nov. is the only known nudibranch species to feed on corals of the genus *Montipora*. Although Rudman (1981: 387) argued feeding on corals cannot count as a true distinguishing feature, evidence suggests prey specialization played a strong role in speciation within Cladobranchia (Goodheart et al. 2017) and the genus *Phestilla* itself (Faucci et al. 2007).

One species, *P. panamica*, did not have any sequences available online so a molecular comparison was infeasible. However, it is clear that *P. panamica* and *Phestilla subodiosus* sp. nov. are not the same species. *P. panamica* grows up to 24 mm, has 18 cerata per row, five precardiac rows, and eight postcardiac rows (Rudman 1982), while the largest observed specimen of *Phestilla subodiosus* sp. nov. was 4 mm (Fig. 2a), had three cerata per row, no precardiac rows, and two postcardiac rows. *Phestilla subodiosus* sp. nov. is also not a juvenile of *P. panamica*, as a 3 mm individual analyzed by Rudman (1982) had three precardiac rows. Furthermore, *P. panamica* and *Phestilla subodiosus* sp. nov. have different coral hosts and live on opposite sides of the Pacific Ocean to our current knowledge Rudman (1982).

There were considerable differences in the reproductive system and radula of *Phestilla subodiosus* sp. nov. and the rest of the genus, notably in the presence of a female gland mass. This arrangement is surprisingly similar to the reproductive system of the Chromodorididae. While it possible that the “female gland mass” is a bursa copulatrix, this would be extremely large for the genus, with a diameter 1.5 times the penile bulb’s length, and directly attached to the female genital opening. In all other species of *Phestilla*, with the exception of *P. chaetopterana* comb. nov., the bursa copulatrix is much smaller than the penile bulb and attached to the oviduct. In *P. chaetopterana* comb. nov., the bursa copulatrix is small, but attached directly to the female genital opening. As the function of the bursa copulatrix’ is to store sperm and/or digest it when needed, a larger one would allow a nudibranch to store more sperm longer thus explaining the phenomena reported by aquarists where the introduction of a single nudibranch can result in an outbreak and their ability to survive long periods without food (D Hui pers. comm. 2018). However, while the specimens dissected for the internal morphology analysis were sexually mature, they were only 2 mm and 3 mm in length. As previously shown, internal morphology has high ontogenetic plasticity throughout development (Ekimova et al. 2019), and further research is required to determine whether the structures recovered represent the final stages of development. Furthermore, in all other *Phestilla* species with the exception of *P. chaetopterana* comb. nov., the denticles extend further than the central cusp, but the cusp of *Phestilla subodiosus* sp. nov. reaches farther than the denticles. The radula of the new species is also the shortest in the genus, with an adult specimen only having 12 teeth, while the next smallest species, *P. minor*, had 30 (Rudman 1981).

## Discussion

While several molecular studies have investigated the phylogenetic relationships within Fionoidae, taxonomic assignment of groups has resulted in debate, including the placement and composition of some genera in Trinchesiidae such as *Phestilla*. Several genera were combined due to their close relationships recovered in a molecular phylogenetic analysis (Cella et al. 2016); however, the absence of synapomorphies led to the reversal of this new classification (Korshunova et al. 2017c). On the same date of the publication as Korshunova et al. (2017c), According to its original description, *Te. chaetopterana* fits within with *Phestilla* morphologically; additionally, our phylogenetic hypothesis found that *Te. chaetopterana* formed a strongly supported clade (96%) with all other *Phestilla* species. We therefore propose reclassifying *Te. chaetopterana* as *Phestilla chaetopterana* comb. nov. Together, *Phestilla subodiosus* sp. nov. and *P. chaetopterana* comb. nov. represent new species that provide clues towards the incomplete puzzle of Fionoidae systematics.

In recent decades, the introduction of coral aquaculture has reshaped both the aquarium industry and coral reef conservation efforts (Cato and Brown 2008; Livengood and Chapman 2007). The ability to culture corals in captivity has fueled the multi-billion-dollar hobbyist industry (Cato and Brown 2008) while relieving collection pressure on natural coral populations (Jones 2011). However, challenges to this technology still exist, including the proliferation of various pests that can damage or kill cultured corals, and are difficult or impossible to eliminate (Bakus 1966; Gochfeld and Aeby 1997; Scott et al. 2017). In particular, *Phestilla* nudibranchs are a problematic group due to their small size and effective camouflage, often evading detection and eradication (Rudman 1979, 1982; Gochfeld and Aeby 1997).

Despite being a prolific pest in aquaria, we were only able to find two reports of nudibranchs that resemble *Phestilla subodiosus* sp. nov. *in situ* (Roberston 1987:3; Cho et al. 2018). However, this seems to be characteristic of *Phestilla* species: their fecundity allows them to decimate entire coral colonies in several days *in vitro* (Fig. 1; Harris 1975; Rudman 1979, 1981, 1982; Haramaty 1991), but their populations are heavily suppressed by predators *in situ* (Gochfeld and Aeby 1997; Mehrotra et al. 2019). It is likely that *Phestilla subodiosus* sp. nov. populations exhibit similar dynamics, and thus are hard to find under natural conditions, likely preventing detection. If this hypothesis is supported, populations of *Phestilla subodiosus* sp. nov. could be controlled in reef tanks through the use of natural predators. Gochfeld and Aeby (1997) identified several fish and crustacean species that preyed on *P. sibogae*. However, further research is required to identify whether these species are also predators of *Phestilla subodiosus* sp. nov. and if they are suitable for a reef aquarium setting. Worthy of note, the outbreak of *Phestilla subodiosus* sp. nov. that led to this description occurred shortly after the death of a *Macropharyngodon meleagris* (Actinopterygii: Labridae) in the aquarium, and another labrid species (*Thalassoma duperrey*) was identified by Gochfeld and Aeby (1997) to feed on *P. sibogae*. *Ma. meleagris* and other labrids may well be suitable candidates for biocontrol of *Phestilla subodiosus* sp. nov.

*Phestilla subodiosus* sp. nov. displayed prey selectivity in our preliminary tests; however, the underlying mechanism is unclear. It has been established that other *Phestilla* species rely on chemical cues to differentiate host corals (Hadfield and Pennington 1990; Kimberly 2003; Ritson-Williams et al. 2009). The extrapolation of this conclusion to *Phestilla subodiosus* sp. nov. is supported by our observations. As *Phestilla subodiosus* sp. nov. ignored all corals except *Montipora* spp. (Table 2), including several that shared the same colony morphology or coenosteum phenotype (Veron 2000; Wallace et al. 2012). We therefore speculate that *Phestilla subodiosus* sp. nov. relies on a non-visual and non-tactile system to identify host colonies, likely chemical cues. Determining how *Phestilla subodiosus* sp. nov. identifies suitable hosts could lead to the development of chemical pest control measures that inhibit these cues.

The description of *Phestilla subodiosus* sp. nov. is a key step that will allow for research to be conducted on its ecology and biology, and eventual control within reef aquaria. Given the wide number of common names in use to describe nudibranchs that feed on *Montipora* spp. (D Hui, J McNelley pers. comm. 2018), it is unclear whether several species exist or whether these names all refer to *Phestilla subodiosus* sp. nov. By formally describing *Phestilla subodiosus* sp. nov., further research can be conducted with confidence in the identity of the species being examined, allowing for clear collaboration and communication while a basic biological and ecological understanding of this species is developed. Furthermore, *Phestilla subodiosus* sp. nov. has been placed on the taxonomic tree of life within a well-understood genus containing several model organisms. Previous studies have described the proteins involved in *Phestilla* metamorphosis and drugs have been discovered that inhibit this vital process (Pires et al. 1997, 2000), providing a potential avenue to control this pest species. However, more research is required to determine if this is a safe and effective method for combating *Phestilla subodiosus* sp. nov. in a reef-aquarium setting.

Despite the scientific advances enabled by the aquarium industry (Veron 2000), this exchange of information and technology has not been reciprocated; hobbyist needs are frequently overlooked by researchers, including research into the control of pests. The earliest digital appearance of the term “*Montipora*-eating nudibranchs” appeared in 2001 (Gray 2001), and it has taken nearly two decades for it to be addressed by the scientific community, illustrating the disconnection between the two groups. The diagnosis of *Phestilla subodiosus* sp. nov. will hopefully pave the way to the control and eradication of a costly pest species in the aquarium industry, and this description presents an example of how collaboration between researchers and aquarists can further both fields.

## Supplementary Material

XML Treatment for Phestilla

XML Treatment for Phestilla subodiosus

## Supplementary materials

Supplementary material 1 Table S1: List of sequences used for molecular analysis Data type: GenBank Accession Numbers Explanation note: The species name and GenBank accessions of sequences we obtained and used for phylogenetic inference, ABGD analysis, p-distance are included within this table. https://binary.pensoft.net/file/376774This dataset is made available under the Open Database License (http://opendatacommons.org/licenses/odbl/1.0/). The Open Database License (ODbL) is a license agreement intended to allow users to freely share, modify, and use this Dataset while maintaining this same freedom for others, provided that the original source and author(s) are credited.Adam Wang, Inga Elizabeth Conti-Jerpe, John Lawrence Richards, David Michael Baker

Supplementary material 2 S2: Phylogenetic hypotheses based upon individual COI, 16s, and H3 partial gene sequences Data type: Newick Trees Explanation note: The compressed file contains 6 newick tree files and are named based upon the partial gene and phylogenetic inference method that were used to generate that tree. BI = Bayesian Inference, ML = Maximum Likelihood. https://binary.pensoft.net/file/376775This dataset is made available under the Open Database License (http://opendatacommons.org/licenses/odbl/1.0/). The Open Database License (ODbL) is a license agreement intended to allow users to freely share, modify, and use this Dataset while maintaining this same freedom for others, provided that the original source and author(s) are credited.Adam Wang, Inga Elizabeth Conti-Jerpe, John Lawrence Richards, David Michael Baker

## References

[B1] Altschul SF Gish W Miller W Myers EW Lipman DJ (1990) Basic local alignment search tool. Journal of Molecular Biology 215(3): 403–410. 10.1016/S0022-2836(05)80360-2 2231712

[B2] Bakus GJ (1966) Some relationships of fishes to benthic organisms on coral reefs. Nature 210(3): 280–284. 10.1038/210280a0

[B3] Bergh LSR (1874) Neue Nacktschnecken der Südsee, Malacologische Untersuchungen II. Journal des Museum Godeffroy 2: 91–116. [pls 1–4] https://biodiversitylibrary.org/page/10860988 [accessed 2 August 2018]

[B4] Borneman E (2007) Two potential molluscicides useful against pest aeolid nudibranchs common on species of *Montipora* in aquariums. Reefkeeping 6(8). http://reefkeeping.com/issues/2007-09/eb/ [accessed 1 July 2018]

[B5] Carmona L Pola M Gosliner TM Cervera JL (2013) A tale that morphology fails to tell: A molecular phylogeny of Aeolidiidae (Aeolidida, Nudibranchia, Gastropoda). PLoS ONE 8 (5): e63000. 10.1371/journal.pone.0063000 PMC3642091 23658794

[B6] Cato J Brown C (2008) Marine Ornamental Species: Collection, Culture & Conservation. Wiley, Iowa, 395 pp.

[B7] Cella K Carmona L Ekimova I Chichvarkhin A Schepetov D Gosliner T (2016) A radical solution: The phylogeny of the nudibranch family Fionidae PLoS ONE 11(23): e0167800. 10.1371/journal.pone.0167800 PMC5158052 27977703

[B8] Chan L Choi L McCorry D Chan K Lee M (2005) Field guide to hard corals of Hong Kong. 郊野公园之友会天地图书有限公司, Hong Kong, 373 pp.

[B9] Chernomor O von Haeseler A Minh BQ (2016) Terrace aware data structure for phylogenomic inference from supermatrices. Systematic Biology 65(6): 997–1008. 10.1093/sysbio/syw037 27121966 PMC5066062

[B10] Cho YG Kang HS Choi JH Choi KS (2018) *Tenellia* sp. YGC-2018 cytochrome c oxidase subunit I gene, partial cds; mitochondrial. GenBank Sequence. http://www.ncbi.nlm.nih.gov/nuccore/MG878397.1 [accessed 2 September 2019]

[B11] Clark K Karsch-Mizrachi I Lipman DJ Ostell J Sayers EW (2016) GenBank. Nucleic Acids Research 44 (Database issue): D67–D72. 10.1093/nar/gkv1276 PMC4702903 26590407

[B12] Colgan DJ McLauchlan A Wilson GDF Livingston SP Edgecombe GD Macaranas J Cassis G Gray MR (1998) Histone H3 and U2 snRNA DNA sequences and arthropod molecular evolution. Australian Journal of Zoology 46(5): 419 10.1071/ZO98048

[B13] Dalton SJ Godwin S (2006) Progressive coral tissue mortality following predation by a corallivorous nudibranch (*Phestilla* sp.). Coral Reefs 25(4): 529–529. 10.1007/s00338-006-0139-0

[B14] Dayrat B Tillier A Lecointre G Tillier S (2001) New clades of euthyneuran gastropods (Mollusca) from 28S rRNA sequences. Molecular Phylogenetics and Evolution 19(2): 225–235. 10.1006/mpev.2001.0926 11341805

[B15] Debelius H Kuiter RH (2007) Nudibranchs of the world: 1,200 nudibranchs from around the world, 1^st^ ed. Ikan, Frankfurt, 360 pp.

[B16] Edgar RC (2004) MUSCLE: multiple sequence alignment with high accuracy and high throughput. Nucleic Acids Research 32(5): 1792–1797. 10.1093/nar/gkh340 15034147 PMC390337

[B17] Ekimova I Deart Y Schepetov D (2017) Living with a giant parchment tube worm: a description of a new nudibranch species (Gastropoda: Heterobranchia) associated with the annelid *Chaetopterus*. Marine Biodiversity 49(1): 289–300. 10.1007/s12526-017-0795-z

[B18] Ekimova I Valdés Á Chichvarkhin A Antokhina T Lindsay T Schepetov D (2019) Diet-driven ecological radiation and allopatric speciation result in high species diversity in a temperate-cold water marine genus *Dendronotus* (Gastropoda: Nudibranchia). Molecular Phylogenetics and Evolution 141(1): 106609 10.1016/j.ympev.2019.106609 31494182

[B19] Faucci A Toonen RJ Hadfield MG (2007) Host shift and speciation in a coral-feeding nudibranch. Proceedings of the Royal Society B: Biological Sciences 274(1606): 111–119. 10.1098/rspb.2006.3685 PMC1679885 17134995

[B20] Folmer O Black M Hoeh W Lutz R Vrijenhoek R (1994) DNA primers for amplification of mitochondrial cytochrome c oxidase subunit I from diverse metazoan invertebrates. Molecular Marine Biology and Biotechnology 3(5): 294–299. 7881515

[B21] Fukami H Omori M Hatta M (2000) Phylogenetic relationships in the coral family Acroporidae, reassessed by inference from mitochondrial genes. Zoological Science 17(5): 689–696. 10.2108/zsj.17.689 18517306

[B22] Gochfeld DJ Aeby GS (1997) Control of populations of the coral-feeding nudibranch *Phestilla sibogae* by fish and crustacean predators. Marine Biology 130(1): 63–69. 10.1007/s002270050225

[B23] Goodheart JA Bazinet AL Valdés Á Collins AG Cummings MP (2017) Prey preference follows phylogeny: evolutionary dietary patterns within the marine gastropod group Cladobranchia (Gastropoda: Heterobranchia: Nudibranchia). BMC Evolutionary Biology 17(1): 221 10.1186/s12862-017-1066-0 29073890 PMC5659023

[B24] Gosliner TM Valdes A Behrens D (2015) Nudibranch and Sea Slug Identification: Indo-Pacific, 1^st^ ed. New World Publications, Jacksonville, FL, 408 pp.

[B25] Gray T (2001) Elimination of a predatory nudibranch. Reeffarmers Articles. http://www.reeffarmers.com/tracygraynudi01.htm [accessed 24 November 2018]

[B26] Hadfield MG Pennington JT (1990) Nature of the metamorphic signal and its internal transduction in larvae of the nudibranch Phestilla sibogae. Bulletin of Marine Science 46(2): 455–464.

[B27] Hadfield MG Faucci A Koehl MAR (2006) Measuring recruitment of minute larvae in a complex field environment: The corallivorous nudibranch *Phestilla sibogae* (Bergh). Journal of Experimental Marine Biology and Ecology 338(1): 57–72. 10.1016/j.jembe.2006.06.034

[B28] Hadfield MG Carpizo-Ituarte EJ Carmen KD Nedved BT (2001) Metamorphic competence, a major adaptive convergence in marine invertebrate larvae. American Zoologist 41(5): 9 10.1093/icb/41.5.1123

[B29] Haramaty L (1991) Reproduction effort in the nudibranch *Phestilla sibogae*: Calorimetric analysis of food and eggs. Pacific Science 45(3): 257–262.

[B30] Harris LG (1975) Studes on the life history of two coral-eating nudibranchs of the genus *Phestilla*. The Biological Bulletin 149(3): 539–550. 10.2307/1540385 29324191

[B31] Henschen B (2018) *Montipora* Eating Nudibranchs | Coral Rx Don’t Risk It Dip It! https://coralrx.com/2018/12/28/montipora-eating-nudibranchs/ [accessed 10 March 2019]

[B32] Hillis DM Moritz C Mable BK (1996) Molecular Systematics, 2^nd^ ed. Sinauer Associates, Inc., Sunderland, Mass, 655 pp 10.2307/1447682

[B33] Hoang DT Chernomor O (2017) UFBoot2: Improving the ultrafast bootstrap approximation. Molecular Biology and Evolution 35(2): 518–522. 10.1093/molbev/msx281 PMC5850222 29077904

[B34] Jones AM (2011) Raiding the coral nurseries? Diversity 3(3): 466–482. 10.3390/d3030466

[B35] Kalyaanamoorthy S Minh BQ Wong TKF von Haeseler A Jermiin LS (2017) ModelFinder: fast model selection for accurate phylogenetic estimates. Nature Methods 14(6): 587–589. 10.1038/nmeth.4285 28481363 PMC5453245

[B36] Kearse M Moir R Wilson A Stones-Havas S Cheung M Sturrock S Buxton S Cooper A Markowitz S Duran C Thierer T Ashton B Meintjes P Drummond A (2012) Geneious Basic: An integrated and extendable desktop software platform for the organization and analysis of sequence data. Bioinformatics 28(12): 1647–1649. 10.1093/bioinformatics/bts199 22543367 PMC3371832

[B37] Kimberly C (2003) Pharmacological and molecular investigations of mechanisms of metamorphosis in the marine gastropod *Phestilla sibogae* PhD Thesis. University of Hawai'i at Mānoa. http://hdl.handle.net/10125/3052

[B38] Korshunova T Martynov A Picton B (2017a) Ontogeny as an important part of integrative taxonomy in tergipedid aeolidaceans (Gastropoda: Nudibranchia) with a description of a new genus and species from the Barents Sea. Zootaxa 4324(1): 1 10.11646/zootaxa.4324.1.1

[B39] Korshunova T Martynov A Bakken T Picton B (2017b) External diversity is restrained by internal conservatism: New nudibranch mollusc contributes to the cryptic species problem. Zoologica Scripta 46(6): 683–692. 10.1111/zsc.12253

[B40] Korshunova T Lundin K Malmberg K Picton B Martynov A (2018a) First true brackish-water nudibranch mollusk provides new insights for phylogeny and biogeography and reveals paedomorphosis-driven evolution. PLoS ONE 13(3): e0192177. 10.1371/journal.pone.0192177 PMC5851531 29538398

[B41] Korshunova T Fletcher K Lundin K Picton B Martynov A (2018b) The genus *Zelentia* is an amphi-boreal taxon expanded to include three new species from the North Pacific and Atlantic oceans (Gastropoda: Nudibranchia: Trinchesiidae). Zootaxa 4482(2): 297–321. 10.11646/zootaxa.4482.2.4 30313822

[B42] Korshunova T Mehrotra R Arnold S Lundin K Picton B Martynov A (2019a) The formerly enigmatic Unidentiidae in the limelight again: A new species of the genus *Unidentia* from Thailand (Gastropoda: Nudibranchia). Zootaxa 4551(5): 556–570. 10.11646/zootaxa.4551.5.4 30790794

[B43] Korshunova T Martynov A Bakken T Evertsen J Fletcher K Mudianta IW Saito H Lundin K Schrödl M Picton B (2017c) Polyphyly of the traditional family Flabellinidae affects a major group of Nudibranchia: aeolidacean taxonomic reassessment with descriptions of several new families, genera, and species (Mollusca, Gastropoda). ZooKeys 717(1): 1–139. 10.3897/zookeys.717.21885 PMC5784208 29391848

[B44] Korshunova T Picton B Furfaro G Mariottini P Pontes M Prkić J Fletcher K Malmberg K Lundin K Martynov A (2019b) Multilevel fine-scale diversity challenges the “cryptic species” concept. Scientific Reports 9(1): 1–23. 10.1038/s41598-019-42297-5 31043629 PMC6494890

[B45] Kumar S Stecher G Li M Knyaz C Tamura K (2018) MEGA X: Molecular Evolutionary Genetics Analysis across computing platforms. Molecular Biology and Evolution 35(6): 1547–1549. 10.1093/molbev/msy096 29722887 PMC5967553

[B46] Le LV Lecointre G Perasso R (1993) A 28S rRNA-based phylogeny of the gnathostomes: First steps in the analysis of conflict and congruence with morphologically based cladograms. Molecular Phylogenetics and Evolution 2(1): 31–51. 10.1006/mpev.1993.1005 8081546

[B47] Livengood EJ Chapman FA (2007) The ornamental fish trade: An introduction with perspectives for responsible aquarium fish ownership. Department of Fisheries and Aquatic Sciences; University of Florida/Institute of Food and Agricultural Sciences: FA123. https://agrilifecdn.tamu.edu/fisheries2/files/2013/10/The-Ornamental-Fish-Trade-An-Introduction-with-Perspectives-for-Responsible-Aquarium-Fish-Ownership.pdf

[B48] Martynov A Mehrotra R Chavanich S Nakano R Kashio S Lundin K Picton B Korshunova T (2019) The extraordinary genus *Myja* is not a tergipedid, but related to the Facelinidae s. str. With the addition of two new species from Japan (Mollusca, Nudibranchia). ZooKeys 818(1): 89–116. 10.3897/zookeys.818.30477 PMC6354008 30723380

[B49] Mehrotra R Monchanin C Scott C Phongsuwan N Caballer Gutiérrez M Chavanich S Hoeksema B (2019) Selective consumption of sacoglossan sea slugs (Mollusca: Gastropoda) by scleractinian corals (Cnidaria: Anthozoa). PLoS ONE 14(4): e0215063. 10.1371/journal.pone.0215063 PMC6488191 31034493

[B50] Morton B Blackmore G Kwok CT (2002) Corallivory and prey choice by *Drupella rugosa* (Gastropoda: Muricidae) in Hong Kong. Journal of Molluscan Studies 68(3): 217–223. 10.1093/mollus/68.3.217

[B51] Nguyen L-T Schmidt HA von Haeseler A Minh BQ (2015) IQ-TREE: A Fast and Effective Stochastic Algorithm for Estimating Maximum-Likelihood Phylogenies. Molecular Biology and Evolution 32(1): 268–274. 10.1093/molbev/msu300 25371430 PMC4271533

[B52] Palumbi SR Martin A Romano S McMillan WO Stice L Grabowski G (2002) The Simple Fool’s Guide to PCR V2.0. Dept. of Zoology and Kewalo Marine Laboratory, University of Hawaii, Hawaii, 45 pp.

[B53] Pasquinelli AE Reinhart BJ Slack F Martindale MQ Kuroda MI Maller B Hayward DC Ball EE Degnan B ller PM Srinivasan A Fishman M Finnerty J Corbo J Levine M Leahy P Davidson E Ruvkun G (2000) Conservation of the sequence and temporal expression of let-7 heterochronic regulatory RNA. Nature 408(6808): 86–89. 10.1038/35040556 11081512

[B54] Pires A Coon SL Hadfield MG (1997) Catecholamines and dihydroxyphenylalanine in metamorphosing larvae of the nudibranch *Phestilla sibogae* Bergh (Gastropoda: Opisthobranchia). Journal of Comparative Physiology. A, Sensory, Neural, and Behavioral Physiology 181(1): 187–194. 10.1007/s003590050105 9309865

[B55] Pires A Croll R Hadfield M (2000) Catecholamines modulate metamorphosis in the opisthobranch gastropod *Phestilla sibogae*. The Biological Bulletin 198(3): 319–331. 10.2307/1542688 10897446

[B56] Puillandre N Lambert A Brouillet S Achaz G (2012) ABGD, Automatic Barcode Gap Discovery for primary species delimitation. Molecular Ecology 21(8): 1864–1877. 10.1111/j.1365-294X.2011.05239.x 21883587

[B57] Puslednik L Serb JM (2008) Molecular phylogenetics of the Pectinidae (Mollusca: Bivalvia) and effect of increased taxon sampling and outgroup selection on tree topology. Molecular Phylogenetics and Evolution 48(3): 1178–1188. 10.1016/j.ympev.2008.05.006 18579415

[B58] Riddle D (2012) Aquarium Invertebrates: *Phestilla* Nudibranchs: Cryptic Enemies of *Porites*, *Goniopora*, *Tubastrea* and *Dendrophyllia* Corals and an Identification of ‘*Montipora*-eating Nudibranchs. Advanced Aquarist 11(6). https://www.advancedaquarist.com/2012/6/inverts

[B59] Ritson-Williams R Shjegstad S Paul V (2003) Host specificity of four corallivorous *Phestilla* nudibranchs (Gastropoda: Opisthobranchia). Marine Ecology Progress Series 255(1): 207–218. 10.3354/meps255207

[B60] Ritson-Williams R Shjegstad SM Paul VJ (2009) Larval metamorphosis of *Phestilla* spp. in response to waterborne cues from corals. Journal of Experimental Marine Biology and Ecology 375(1): 84–88. 10.1016/j.jembe.2009.05.010

[B61] Robertson R (1970) Review of the Predators and Parasites of Stony Corals, with Special Reference to Symbiotic Prosobranch Gastropods. Pacific Science 24(1): 43–54.

[B62] Ronquist F Teslenko M van der Mark P Ayres DL Darling A Höhna S Larget B Liu L Suchard MA Huelsenbeck JP (2012) MrBayes 3.2: Efficient bayesian phylogenetic inference and model choice across a large model space. Systematic Biology 61(3): 539–542. 10.1093/sysbio/sys029 22357727 PMC3329765

[B63] Rudman WB (1979) The ecology and anatomy of a new species of aeolid opisthobranch mollusc; a predator of the scleractinian coral *Porites*. Zoological Journal of the Linnean Society 65(4): 339–350. 10.1111/j.1096-3642.1979.tb01099.x

[B64] Rudman WB (1981) Further studies on the anatomy and ecology of opisthobranch molluscs feeding on the scleractinian coral *Porites*. Zoological Journal of the Linnean Society 71(4): 373–412. 10.1111/j.1096-3642.1981.tb01136.x

[B65] Rudman WB (1982) A new species of *Phestilla*; the first record of a corallivorous aeolid nudibranch from tropical America. Journal of Zoology 198(4): 465–471. 10.1111/jzo.1982.198.4.465

[B66] Scott C Mehrotra R Hoeksema B (2017) In-situ egg deposition by corallivorous snails on mushroom corals at Koh Tao (Gulf of Thailand). Journal of Molluscan Studies 83(3): 360–362. 10.1093/mollus/eyx020

[B67] Sela I Ashkenazy H Katoh K Pupko T (2015) GUIDANCE2: accurate detection of unreliable alignment regions accounting for the uncertainty of multiple parameters. Nucleic Acids Research 43(W1): W7–W14. 10.1093/nar/gkv318 PMC4489236 25883146

[B68] Veron C (2000) Corals of the World. Australian Institute of Marine Science, 1382 pp.

[B69] Wägele H Willan RC (2000) Phylogeny of the Nudibranchia. Zoological Journal of the Linnean Society 130(1): 83–181. 10.1111/j.1096-3642.2000.tb02196.x

[B70] Wallace CC Done BJ Paul RM (2012) Revision and catalogue of world-wide staghorn corals *Acropora* and *Isopora* (Scleractinia:Acroporidae) in the Museum of Tropical Queensland. Memoirs of the Queensland Museum Nature 57: 255.

