# Supplementary material for: Phestilla subodiosus sp. nov. (Nudibranchia, Trinchesiidae), a corallivorous pest species in the aquarium trade
Source: Zookeys. 2020 Feb 5;909:1–24. doi: 10.3897/zookeys.909.35278 (PMC7015969; doi:10.3897/zookeys.909.35278)
Supplement: Supplementary material 1 [file zookeys-909-001-s001.docx]

| **Nudibranch Species** | **COI** | **16S** | **H3** | **28S** |
| --- | --- | --- | --- | --- |
| **Phylogeny** | | | | |
| *Calma glaucoides* | MG323544 | MG323550 | MG323565 |  |
| *Catriona columbiana* | KY128906 | KY128698 | KY128493 |  |
| *Catriona* cf. *maua* | KY128905 | KY128697 | KY128492 |  |
| *Catriona gymnota* | KY128907 | KY128699 | KY128494 |  |
| *Cuthona nana* | KY128962 | KY128755 | KY128549 |  |
| *Cuthonella cocoachroma* | KY128925 | KY128720 | KY128513 |  |
| *Cuthonella concinna* | KY128924 | KY128719 | KY128512 |  |
| *Cuthonella hiemalis* | KY129007 | KY128799 | KY128595 |  |
| *Cuthonella soboli* | KY129020 | KY128811 | KY128605 |  |
| *Cuthonella* sp. A | KY129019 | KY128810 | KY128594 |  |
| *Diaphoreolis flavovulta* | KY128947 | KY128742 | KY128535 |  |
| *Diaphoreolis lagunae* | KY128955 | KY128748 | KY128542 |  |
| *Diaphoreolis viridis* | KY129104 | KY128898 | KY128690 |  |
| *Eubranchus alexeii* | KY128900 | KY128692 | KY128487 |  |
| *Eubranchus* cf. *farrani* | KY129028 | KY128819 | KY128614 |  |
| *Eubranchus exiguous* | KY129029 | KY128820 | KY128615 |  |
| *Eubranchus farrani* | KY513666 | KY513644 | KY513685 |  |
| *Eubranchus mandapamensis* | KY129035 | KY128826 | KY128621 |  |
| *Eubranchus odhneri* | KY128903 | KY128695 | KY128490 |  |
| *Eubranchus olivaceus* | KY129037 | KY128828 | KY128623 |  |
| *Eubranchus pallidus* | KY129030 | KY128821 | KY128616 |  |
| *Eubranchus rupium* | KY129038 | KY128829 | KY128624 |  |
| *Eubranchus rustyus* | KP871641 | KP871689 | KP871665 |  |
| *Eubranchus* sp. 3 | KY129040 | KY128831 | KY128626 |  |
| *Eubranchus* sp. A | KY129039 | KY128830 | KY128625 |  |
| *Eubranchus tricolor* | KY129032 | KY128823 | KY128618 |  |
| *Fiona pinnata* | KY129047 | KY128838 | KY128486 |  |
| *Murmania antiqua* | KY129066 | KY128857 | KY128651 |  |
| *Phestilla chaetopterana* comb. nov. | MF458314 | MF458308 | MF458310 |  |
| *Phestilla lugubris* | KY129075 | KY128866 | KY128660 |  |
| *Phestilla melanobrachia* | KY129076 | KY128867 | KY128661 |  |
| *Phestilla poritophages* | KY128969 | KY128760 | KY128555 |  |
| *Phestilla subodiosus,* sp. nov. PS1 | MN255477 | MN255475 | MN255482 |  |
| *Phestilla subodiosus,* sp. nov. PS3 | MN255478 | MN255476 | MN255484 |  |
| *Rubramoena amoena* | KY128904 | KY128696 | KY128491 |  |
| *Rubramoena rubescens* | KY128916 | KY128710 | KY128503 |  |
| *Tenellia adspersa* | KY129085 | KY128876 | KY128668 |  |
| *Tenellia* sp. 2 | KY128985 | KY128777 | KY128572 |  |
| *Tenellia* sp. 3 | KY129080 | KY128871 | KY128665 |  |
| *Tenellia* sp. A | KY128992 | KY128784 | KY128579 |  |
| *Tenellia* sp. J | KY129103 | KY128894 | KY128686 |  |
| *Tenellia* sp. L | KY129074 | KY128865 | KY128659 |  |
| *Tenellia* sp. M | KY128995 | KY128787 | KY128582 |  |
| *Tergeipes tergipes* | KY129087 | KY128878 | KY128670 |  |
| *Teriposacca longicerata* | KY129086 | KY128877 | KY128669 |  |
| *Trinchesia foliata* | KY128912 | KY128704 | KY128499 |  |
| *Trinchesia speciosa* | KY128998 | KY128790 | KY128585 |  |
| *Zelentia* cf. *pustulata* | KY128972 | KY128764 | KY128559 |  |
| *Zelentia fulgens* | KY128951 | KY128746 | KY128539 |  |
| **Sequences Obtained** | | | | |
| *Phestilla subodiosus,* sp. nov. PS1 | MN255477 | MN255475 | MN255482 | MN449477 |
| *Phestilla subodiosus,* sp. nov. PS2 |  |  | MN255483 | MN449478 |
| *Phestilla subodiosus,* sp. nov. PS3 | MN255478 | MN255476 | MN255484 |  |
| *Phestilla subodiosus,* sp. nov. PS4 | MN255479 |  | MN255485 | MN449479 |
| *Phestilla subodiosus,* sp. nov. PS5 | MN255480 |  | MN255486 | MN449480 |
| *Phestilla subodiosus,* sp. nov. PS6 | MN255481 |  | MN255487 |  |
| **Sequences used for Automatic Barcode Gap Discovery and Pairwise distance (*p*-distance) calculation** | | | | |
| *Phestilla subodiosus,* sp. nov. PS1 | MN255477 |  |  |  |
| *Phestilla subodiosus,* sp. nov. PS3 | MN255478 |  |  |  |
| *Phestilla subodiosus,* sp. nov. PS4 | MN255479 |  |  |  |
| *Phestilla subodiosus,* sp. nov. PS5 | MN255480 |  |  |  |
| *Phestilla subodiosus,* sp. nov. PS6 | MN255481 |  |  |  |
| *Phestilla minor* | DQ417301 |  |  |  |
| *Phestilla sibogae* | DQ417287 |  |  |  |
| *Phestilla* sp. 2 | DQ417286 |  |  |  |
| *Phestilla chaetopterana* | MF458313 |  |  |  |
| *Phestilla lugubris* | KY129075 |  |  |  |
| *Phestilla melanobrachia* | KY129076 |  |  |  |
| *Phestilla poritophages* | KY128968 |  |  |  |
| *Phestilla* sp. L | KY129074 |  |  |  |
| *Phestilla* sp. 1 | KU971235 |  |  |  |
| *Phestilla* sp. 3 | MG878397 |  |  |  |
